# Viral hemorrhagic fevers in the Tihamah region of the western Arabian Peninsula

**DOI:** 10.1371/journal.pntd.0005322

**Published:** 2017-04-06

**Authors:** Fathiah Zakham, Mohammed Al-habal, Rola Taher, Altaf Alaoui, Mohammed El Mzibri

**Affiliations:** 1 Department of Laboratory Medicine, Faculty of Medicine and Health Sciences, Hodeidah University, Hodeidah, Yemen; 2 Epidemiological Surveillance Administration, Office of Public Health and Population, Hodeidah, Yemen; 3 Laboratory of Engineering Sciences and Modeling, Ibn Tofail University, Kenitra, Morocco; 4 Biology and Medical Research Unit, Life Sciences Division, CNESTEN, Rabat, Morocco; University of Queensland, AUSTRALIA

## Abstract

Viral hemorrhagic fever (VHF) refers to a group of diseases characterized by an acute febrile syndrome with hemorrhagic manifestations and high mortality rates caused by several families of viruses that affect humans and animals.

These diseases are typically endemic in certain geographical regions and sometimes cause major outbreaks. The history of hemorrhagic fever in the Arabian Peninsula refers to the 19th century and most outbreaks were reported in the Tihamah region—the Red Sea coastal plain of the Arabian Peninsula in the west and southwest of Saudi Arabia and Yemen.

Herein, we describe the agents that cause VHFs and their epidemiology in Tihamah, the history of the diseases, transmission, species affected, and clinical signs. Finally, we address challenges in the diagnosis and control of VHFs in this region.

## Background

Viral hemorrhagic fever (VHF) refers to a group of emerging diseases characterized by acute febrile syndromes with vascular damages, hemorrhagic manifestations, and high mortality rates [1]. Some VHF agents could cause an outbreak of a febrile illness in 2 to 21 days after infection. Specific signs and symptoms vary according to the infecting virus, but initial signs and symptoms often include noticeable fever, fatigue, dizziness, muscle aches, loss of strength, vomiting, and diarrhea [2]. Coagulation defects can lead to more severe clinical symptoms that include bleeding under the skin, causing petechiae, and sometimes conjunctivitis in the eye. Bleeding may also occur in internal organs and from orifices. Despite widespread bleeding, blood loss is rarely the cause of the death [2]. VHFs may also induce thoracic manifestations, including pleural effusion, pneumonia, pulmonary hemorrhage and hemoptysis, acute respiratory distress syndrome, and uncommon manifestations such as acute kidney injury [3, 4].

The VHFs include infections caused by viruses of the families Flaviviridae (dengue fever, yellow fever, Omsk hemorrhagic fever, Kyasanur Forest disease, Alkhurma hemorrhagic fever [AHF]), Bunyaviridae (Crimean-Congo hemorrhagic fever [CCHF], Rift Valley fever [RVF], and Hantavirus diseases), Arenaviridae (Argentine, Bolivian, Brazilian, and Venezuelan hemorrhagic fevers and Lassa fever), and Filoviridae (Ebola and Marburg hemorrhagic fevers) [5]. These diseases are all caused by RNA viruses enveloped in a lipid bilayer coating derived from the host cell membrane [2]. The persistence of these viruses in nature depends on a natural reservoir host, which is an animal or an insect. Some of these viruses may be transmitted from person to person (Lassa, Crimean Congo hemorrhagic fever, Ebola, Marburg, and several other viruses) by direct contact with blood or other body fluids of infected patients [2].

Confirmation of viral etiology is guided by the epidemiological setting simultaneously with research for other microbial or parasitic agents causing hemorrhagic fever syndromes. These diseases are typically endemic in certain geographical regions and sometimes cause major outbreaks [5]. The history of hemorrhagic fever in the Arabian Peninsula refers to the 19th century and the first recorded outbreak of a dengue-like disease, which occurred from 1870 to 1873 [6–8]. After the mid-1990s, the reported outbreaks in the Arabian Peninsula raised the possibility of widespread emergence of VHFs in the region[9]. Most recorded outbreaks were in the Tihamah region—the Red Sea coastal plain of the Arabian Peninsula in the west and southwest of Saudi Arabia and Yemen, and the frequency of such outbreaks has risen enormously in the last two decades [10, 11].

In this review, we outline the situation of VHFs in the Tihamah region of both countries and describe the causative agents and their epidemiology in the region. We discuss the history of the diseases, their transmission, species affected, and clinical signs.

Finally, we address the challenges of the diagnosis and control measures for VHFs in this region to draw the attention of public health authorities in both countries and international health organizations to the alarming situation of VHFs and their dissemination in the region and to stimulate a prompt action towards the management of VHFs in Tihamah.

## Methodology

A literature review was conducted by the authors in December 2015 using electronic databases, including Pubmed/Medline, Science Direct, Google, and Google Scholar. Peer-reviewed literature from 1984 to 2016 was retrieved based on the following keywords: “hemorrhagic fever,” ‘‘Tihamah” or “Tihama,” and “hemorrhagic fever in Saudi Arabia, Yemen, and Arabian Peninsula.” Databases from the World Health Organization (WHO, http://www.who.int) and a report from the electronic disease early warning system in Yemen (eDEWS) were also consulted. For the demographic features of Tihamah, the authors referred to the latest census reports of the General Authority for Statistics in Saudi Arabia and the Central Statistical Organization of Yemen [12, 13].

Only one publication in French was included, while all others are in English. A total of 59 references were reviewed to figure out our conclusion.

## Geographic, climatic and demographic features of Tihamah

**Fig 1:**
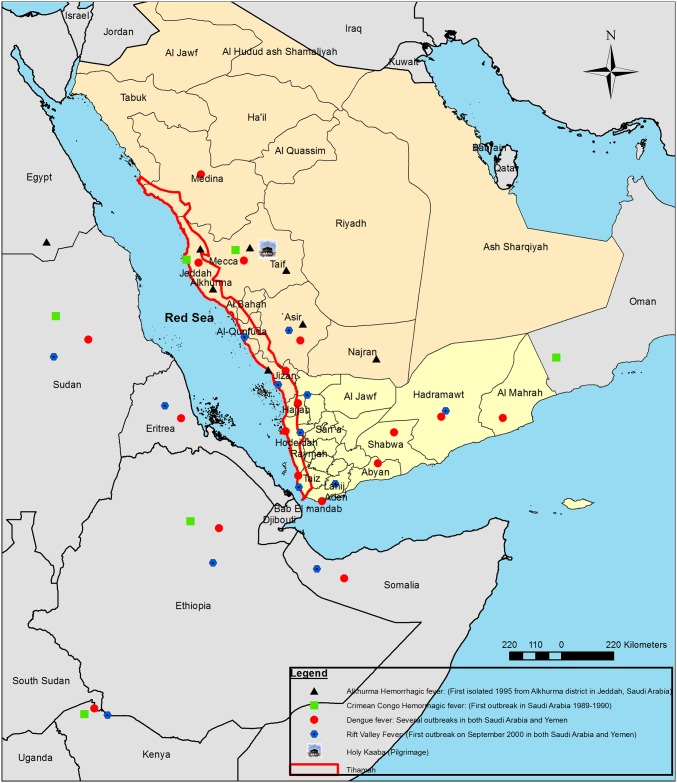
Map of Tihamah in Saudi Arabia and Yemen showing the main identified Viral Hemorrhagic Fevers (VHFs) in the region, adjoining regions and neighboring countries.

Tihamah or Tihama refers to the Red Sea coastal plain of the Arabian Peninsula. The region contains two parts: Tihamah of Al-Hejaz (northern part) and Tihamah Asir, extending to the southern part of the peninsula and reach to Bab el Mandeb in Yemen (Fig 1).

The plain is constricted and extents its greatest widths (60 to 80 km) in the south of Medina and Mecca. The region consists of low hillocks adjoining the north–south Sarawat mountain chain and includes valleys that emerge from the mountains [14]. The Tihamah of Saudi Arabia includes five main regions: small parts of Medina, Mecca, Al Bahah, Asir, and the whole part of Jizan. Each region is divided into a number of governorates and districts, many of them are vastly interconnected with other adjoining regions. In Yemen, three main governorates are within the Tihamah region, which includes small parts of both Hajah and Taiz and the whole city of Hodiedah. Each governorate is divided into different districts (Table 1). Hodeidah is the largest Tihami part and it includes 26 districts.

**Table 1:** Population statistics in Tihamah region.

Regions	Population	Population Density* (per km^2^)	Last Census
Medina (Yunbu and Badr)	298,675	9.9	2010 [12]
Mecca (including Rabigh, Jeddah, Mecca, Al-lith and Qunfuda)	5,636,886	37.9	2010 [12]
Al Bahah (Al mukhwah and Qilwah)	129,568	38.1	2010 [12]
Asir (Abha, Muhayil, Rijal Alma and Al Majarda)	771,631	22.0	2010 [12]
Whole region of Jizan	1,374,845	101.6	2010 [12]
Hajah (Medi, Harad, Haran and Abs)	259,442	186.1	2013 [13]
Whole governorate of Hodeidah	3,774,914	215.5	2013 [13]
Taiz (Al Mokha and Thobab)	80,586	25.31	2013 [13]

*The population density for the whole region is considered.

Due to the availability of water sources for cultivation over the last three centuries, the Tihamah ecological habitat has undergone extensive agricultural development. These changes have a direct impact on the environment, making it more favorable for the virus’ mosquito vectors [14]. The current methods of irrigation in the region depend mainly on the spate flow, and together with the rainfall, this results in many large and small water pools suitable as breeding sites for certain mosquito species and increases the transmission of viral agents [14]. The climate of the Tihamah region is hot, windy, and tropical arid to semiarid. Average temperatures range from 24°C in the winter to more than 35°C in the summer. Rainfall varies from 50 mm in the arid coastal strip to about 350 mm near the foothills, and the evaporation is high (2,400–3,000 mm) and relative humidity is very high, especially during the night and morning, varying from 50% to 70% [15].

The population of Tihamah is one of the highest regions in the Arabian Peninsula, with population density reaching to 215.5 per km2 (in Hodeidah). The total population of the Tihamah part in Saudi Arabia is around 8,211,605, according to the reports of the 2010 census of the General Authority for Statistics in Saudi Arabia; however, the population density varies in the main five regions (Table 1). In Yemen, the population of Tihamah is estimated by the Central Statistical Organization of Yemen to be 4,114,942 in 2013, and its population density is the highest among all the other regions of Tihamah [12, 13].

The region also has different seaports: Jeddah (in the region of Mecca), Jizan, and Hodeidah. Tihamah is an important trade route from Asia to Africa and there is a remarkable African influence in the region. The rural economy depends mainly on agriculture and pastoralism, which represent the potential food security in both countries [15].

The lawlessness on Yemeni borders increased the number of African refugees coming from African horn countries. Moreover, the current war in Yemen raised the number of Yemeni refugees from other regions.

## Hemorrhagic fever viruses circulating in Tihamah

Several cases and outbreaks caused by viruses of the families Flaviviridae and Bunyaviridae have been recorded in the Arabian Peninsula, but there is no evidence for the presence of Arenaviridae and Filoviridae species in this region. The most common viruses in western Saudi Arabia are dengue, Rift Valley fever, Alkhurma hemorrhagic fever, and Crimean-Congo hemorrhagic fever viruses (CCHFV) [16]. In Yemen, the only described viruses are dengue and Rift Valley fever viruses. Here, we highlight the documented viruses in Tihamah.

### Dengue virus

Dengue is the most common arbovirus infection that belongs to the family Flaviviridae, genus *Flavivirus*, and can be transmitted by the bite of infective female mosquitoes of the species *Aedes aegypti* and, to a lesser extent, *A*. *albopictus* [17]. There are four serotypes dengue virus (DENV1–4), but infection with one of them does not provide cross-protective immunity against the other serotypes [17–19].

After an incubation period of two to five days, dengue virus may cause a mild flu-like illness or quickly progresses to serious dengue hemorrhagic fever–dengue shock syndrome [20, 21].

Alarmingly, the incidence of dengue fever increased dramatically between 1990 and 2013, from 8.3 million cases in 1990 to 58.4 million cases in 2013[22].

The history of dengue-like virus in Arabian peninsula dates back to 1870–1873 during an outbreak in Tanzania when the diseases known as dinga, dyenga, or dengue, then spread to several countries in East Africa, Saudi Arabia (Jeddah, Mecca, and Medina), and Yemen (Aden) [8]. A new pandemic was recorded in Yemen from 1907 to 1913 and from 1920 to 1926 in Aden [8, 23].

In 1954, an epidemic of dengue affected 98% of inhabitants in Hodeidah, Yemen [7, 8]. The first serological confirmation from Hodeidah was in 1984, when a traveler returning from Yemen to the United States was serologically diagnosed with dengue [24]. Currently, dengue outbreaks have been documented in Saudi Arabia, mainly in Jeddah (1994–2006) and Mecca (2004) and in Yemen (2000–2006) [20, 25–28].

Most recently, an Italian patient who had returned from Yemen to Italy in 2010 was confirmed to have dengue virus infection due to serotype DENV3 [29]. The endemicity of this disease is also confirmed in the south-western part of Saudi Arabia, mainly in Jizan and Asir [30, 31].

In 2012, an outbreak reported the cocirculation of dengue virus and Chikungunya virus (CHIKV) in different districts of Hodeidah governorate. The predominant serotype was DENV2 [10, 32]. Dengue fever and dengue hemorrhagic fever are frequent public health problems in both countries and the most frequently isolated serotypes in the region are DENV1–3 [9, 26].

### Alkhurma hemorrhagic fever virus

Alkhurma hemorrhagic fever virus (AHFV) is a tick-borne encephalitis *Flavivirus* (family: Flaviviridae) that can be transmitted by the bite of soft *Ornithodoros savigni* and hard *Hyalomma dromedarii* ticks collected from camels and sheep [33]. It was first isolated in Saudi Arabia and described as a unique viral agent of Arabian Peninsula [6, 34]. Significantly, AHFV shares a high similarity with Kyasanur Forest disease virus, which was isolated in India [35].

After an incubation period of two to four days, the disease presents initially with nonspecific influenza like symptoms, including fever, anorexia, malaise, diarrhea, and vomiting. A second phase includes neurologic and hemorrhagic symptoms in severe form. Multiorgan failure leads to fatal outcomes. AHF is a zoonotic disease and clinical cases have been attributed to exposure to livestock (camels and sheep), but AHFV is not isolated yet from such animals [34].

It was isolated in 1995 in Jeddah from six patients in Alkhurma district [36]. Since its first description, several hundred cases of AHF have been reported in different western Saudi governorates with cases peaking in spring and summer [35].

From 2001 to 2003, AHFV was identified by Madani et al. in Mecca [37]. Consequently, several sporadic cases were recorded in Najran from 2003 to 2009 [38].

A recent retrospective study in Saudi Arabia of all laboratory confirmed cases of AHF collected from 2009 to 2011 showed a high percentage of cases in the Najran region.

Najran is an agricultural zone with emphasis on livestock farming, suggesting that cases may have arisen from contact with animals [33]. A second possibility could be the improved surveillance in this region compared to other regions such as Mecca, Jizan, Taif, and Asir, which have similar agricultural conditions [33].

There are no reported cases of AHF in Yemen, but due to the large geographic range of the tick vector, it is expected that the distribution of the virus is expanding, with likely undetected human cases in Yemen [35]. In 2010, the virus was detected in two Italian travelers coming from the southern part of Egypt, which affirms an overseas expansion of geographic distribution of AHFV [39].

### Crimean-Congo hemorrhagic fever virus

CCHF is caused by infection with a tick-borne virus *Nairovirus* within the family Bunyaviridae. It is the most widespread tick‐borne viral infection of humans and the second most widespread of all medically important arboviruses after dengue viruses. CCHFV causes a subclinical disease in most livestock species and is maintained in the herds through the bite of ixodid ticks of the genus *Hyalomma* [40].

The length of the incubation period depends on the mode of transmission of the virus. If the infection is influenced by a tick bite, the incubation period is usually one to five days. The incubation period following contact with infected blood or tissues is usually 5–7 days, with a documented maximum of 13 days [40, 41].

The first signs of disease are not specific (prehemorrhagic phase). The hemorrhagic phase is characterized by a petechial rash of the skin, conjunctiva, and other mucous membranes, which progresses to bleeding from the gastrointestinal and urinary tracts. Hepato- and splenomegaly are common. As a result of hemorrhage, multiorgan failure and shock can occur [40].

CCHF virus was first recognized in Crimea in southeastern Europe on the northern coast of the Black Sea after the Nazi invasion in the mid-1940s and named Crimean hemorrhagic fever [41, 42]. Then, it was isolated in Congo (present Democratic Republic of the Congo) in 1969, thus resulting in the name of the disease. Currently, CCHF is endemic in many countries in Africa, Europe, and Asia [43].

Outbreaks of CCHF have also been reported in western Saudi Arabia (1989–1990) [20, 44]. It was suspected that the CCHF virus was introduced to Saudi Arabia by infected ticks on imported livestock (camels, cattle, sheep, goats, and buffaloes), arriving to the Jeddah seaport. The main risk factor was the exposure to the blood or tissue of livestock in abattoirs, but not tick bites [44]. In 1997, a serosurvey for CCHF antibody was conducted on humans and imported livestock in Saudi Arabia and clearly showed that Sudanese small ruminants exhibited the highest antibody prevalence among all imported livestock (4.25%) [45]. An isolation trails from ticks on Sudanese sheep arrived to Jeddah via the seaport in 1995 demonstrated the presence of CCHF virus in some of those ticks [46].

The CCHF is related to the scarification during the Eid-al-Adha feast with lack of controlling livestock movements in and between countries [47]. Despite the proximity between Yemen, Saudi Arabia, and the African horn, there are no registered cases of CCHF in Yemen neither in human nor animals.

### Rift Valley fever virus

RVF is a vector-borne zoonotic disease caused by a *Phlebovirus* (family Bunyaviridae) [48]. RVF virus is transmitted by *Culex* and *A*. *aegypti* mosquitoes, resulting in large epizootics in livestock, which causes abortion in pregnant ruminants and rapid death in neonates [49]. Humans are incidentally infected when they are bitten by infected mosquitoes, come into contact with aborted or infected animal tissues, or drink unpasteurized milk. It is also a potential bioterrorism agent [49]. Humans suffering from RVF experience influenza-like symptoms that after the initial febrile stage, in some cases, can develop into hemorrhagic fever, encephalitis, and death [49].

RVF was historically restricted to the African continent, but a recent outbreak occurred on the Arabian Peninsula [43, 49–53]. In 2000, RVF virus caused two simultaneous outbreaks in Yemen and Saudi Arabia, mainly in the Tihamah region [50, 54]. On September 15, 2000, the US Centers for Disease Control and Prevention (CDC) isolated RVF virus from Saudi Arabia, and five days after the outbreak of Saudi Arabia, Yemen officially declared its first outbreak of RVF [15]. Saudi Arabia reported 882 human cases and 124 deaths. In Yemen, 1,328 cases and 166 deaths were reported [20, 51].

The viruses from Saudi Arabia and Yemen were almost identical to those associated with earlier RVF epidemics in East Africa, and RNA sequences analysis showed similar phylogenetic relationships among these viruses [55].

In Saudi Arabia and Yemen, the ecosystem structure of the RVF-affected Tihamah regions (western Rift Valley zone) is similar to that of the west floor of the Rift Valley in Ethiopia and Eritrea, across the Red Sea [14]. Recurrence of outbreaks is now documented in Egypt and the Arabian Peninsula [50].

Furthermore, the isolated RFV viruses from the potential mosquito vector in Tihamah, *A*. *vexans arabiensis*, were closely related to the isolated strains in Madagascar and Kenya from 1991 to 1997. This finding suggests the transmission of the virus was from imported mosquitos or livestock from East Africa [52, 56].

A recent seroepidemiological study in the southwestern regions of Saudi Arabia (Jizan, Asir, and Al-Qunfuda) revealed the lack of recent RVF activity among humans in the study areas since the outbreak of 2000 [57].

## Diagnosis and disease control challenges

Through the limited numbers of published data about the outbreaks of VHFs that occurred in the Tihamah region, this review addressed the real threat of those illnesses in the community, especially in the Arabian Peninsula. The first description of VHFs in Tihamah was in the last 19th century, and after the mid-1990s, the outbreaks of several VHFs appeared and drew special attention of health care providers from the entire world.

Most of those epidemics and epizootics were well characterized before their last introduction in the Arabian Peninsula except the Alkhurma virus, which is the only hemorrhagic fever virus isolated for the first time in the Arabian Peninsula.

Most of those outbreaks were in the African continent and mainly in the neighboring African horn countries (Somalia, Ethiopia, Djibouti, and Eritrea) and Sudan (Fig 1). Furthermore, the southern part of the Arabian Peninsula (Bab el Mandeb) has a very close position to the African horn and a long story of animal trade and human movements. Tihamah is an entry gate for animal trade heading towards the other parts of the Arabian Peninsula [15]. Significantly, the recorded VHFs were arthropod-borne disease and mainly mosquito-borne diseases rather than tick-borne diseases, especially in Yemen.

Therefore, the early detection and diagnosis are mandatory to the control of hemorrhagic fever diseases in Tihamah.

Encompassing the spread of such diseases in a consistent community requires an active observation for signs of an outbreak, rapid declaration of its frequency, diagnosis, and identification of the causal agent, in addition to strategies and resources for an appropriate and efficient response.

Although the responses are often observed in terms of human and animal public health, there are also challenges limiting the control of these diseases in the region of Tihamah, especially, with the lack of vaccine and treatment.

Diagnosis may be delayed due to clinicians' unfamiliarity with these diseases and lack of widely available diagnostic tests, or even differential diagnosis reagents, in some health care institutions.

Moreover, the clinical microbiology and public health laboratories in Yemen are not currently equipped to make a rapid diagnosis of these viruses, and clinical specimens in a new outbreak need to be sent to the regional WHO office and the CDC.

Laboratory detection of viruses is based on the detection of viral antigen and IgM/IgG antibodies, usually by ELISA (which is not always available) or by other serological rapid tests. Indeed, this is not enough for the detection of viral genome and makes the isolation of new viruses either ambiguous or underestimated, like the case of Alkhurma virus. Additionally, Tihamah is an endemic area for several tropical illnesses, and the contributing symptoms may lead to misdiagnosis in complicated cases.

For VHFs that are spread by arthropod vectors, prevention efforts should focus on community-wide arthropod control, which poses a great challenge in Tihamah.

Yemen has the highest percentage of people living in poverty of all the Middle East/North African countries with high rates of neglected tropical diseases [58].

In addition to poverty, political conflict in Yemen and associated breakdowns in public health and livestock control have contributed to the emergence of these infections. Furthermore, the most affected region (Tihamah) lies on the Red Sea coast with movement of refugees from and to the African horn [10].

Moreover, the water insufficiency and lack of infrastructure in urban and rural areas require storage of water in water containers and tanks, which create a good environment for mosquitos [10, 32]. The sewers overflow in the port city of Hodeidah and electrical outages have aggravated the situation during the current war. Owing to all those factors, breeding sites of mosquitoes have increased, making the region a favorable habitat for their reproduction.

Worldwide, fumigation campaigns are very useful for controlling vectors; however, the most accessible mitigation in almost all Yemeni governorates is still based on mass spraying, while the distribution of vectors can be disturbingly more heterogeneous. Indoor residual spray (IRS) has been increasingly used and considered to be a potential alternative to mass spraying. The use of the IRS should be an essential part of the vector control strategies where this intervention is appropriate. According to the latest reports from the eDEWS in Yemen, residual fumigation campaigns have been recently adopted in some regions, including Hodeidah, but an extension of response is needed for a good prevention program [59].

Likewise, in southwestern Saudi Arabia, several factors could lead to human infection with these viruses, including the lack of electricity, having livestock in the house, slaughtering animals, and contact with or transporting aborted and dead animals [57].

Saudi Arabia annually hosts 2–3 million people from across the world for the pilgrimage (Hajj). The Hajj rituals require sacrifice of livestock (more than 10–15 million small ruminants annually), and their importation from other countries may contribute to the global spread of those pathogens [6, 11].

Additionally, the widespread agricultural development in Tihamah, the use of modern irrigation technologies such as spate flow and rainfall, and the hot/humid weather in the area contribute to breeding sites for vectors, especially mosquitos. All these factors are thought to be the reasons for the emergence of VHFs in the region, threatening the new adjoining ecozones like Najran, Taiz, and other southern parts of the Arabian Peninsula, mainly in Yemen [14].

Patients infected with VHF should receive supportive therapy, with special attention to maintaining fluid and electrolyte balance, circulatory volume, blood pressure, and treatment for complicating infections. This is not always possible in Yemen due to the inadequate health care infrastructure, especially in rural areas. Additionally, reporting of cases to health authorities is not always accurate and could be misleading.

The lack of community awareness due to illiteracy or inadequate education also represents a great problem in the affected region. Thus, emphasis on educating and empowering individuals and communities should be the first action considered in the fight against VHFs. The dissemination of information among health care professionals and within communities is also a critical for the achievement of these goals.

Better management of VHFs in Tihamah depends on the implementation of general strategies and policies at different levels.

At the individual level, scientists, researchers, and health care providers should build strong competencies to assess the needs of the community, find suitable solutions, and develop strategies for better response and control of VHF epidemics. Increasing the awareness and interactivity within the society and providing training on clinical management, diagnosis, and vector control at the local level with different collaborative centers are essential activities for the effective management and preventive action.

At the national level, there is an urgent need to reinforce the disease surveillance and notification system in collaboration with public health authorities to stimulate prompt public health action. The setup of sentinel surveillance centers could provide timely information on the disease situation, which serves as an indicator of the quality of the local preventive or therapeutic measures.

At the international level, health care agencies, organizations, and governments should strive for a deep understanding of those diseases. Collaboration between nations is the most effective strategy for overcoming challenges and enhancing public health surveillance in and between countries.

**Key learning points**

- Viral hemorrhagic fever (VHF) diseases and outbreaks in the region of Tihamah are considered a serious public health concern, threatening the life of humans and animals.
- The most circulating viruses in Tihamah are dengue, Rift Valley, Alkhurma, and Crimean-Congo hemorrhagic viruses.
- The geographic, climatic, and demographic features of Tihamah have an extreme impact on the epidemics of VHFs.
- The establishment of a sustainable and reliable disease surveillance system is very important for the control and prevention of those illnesses.

**Key papers**

1. Van Kleef E, Bambrick H, Hales S (2011). The geographic distribution of dengue fever and the potential influence of global climate change. TropIKA Reviews (TropIKAnet): 22.
2. Glyn Davies F, Martin V (2003) Recognizing Rift Valley fever—Food and Agriculture. Rome: Food and Agriculture Organization of the United Nations.
3. Ciccozzi M, Lo Presti A, Cella E, Giovanetti M, Lai A, et al. (2014) Phylogeny of Dengue and Chikungunya viruses in Al Hudayda governorate, Yemen. Infection, Genetics and Evolution 27: 395–401.
4. Balkhy HH, Memish ZA (2003) Rift Valley fever: an uninvited zoonosis in the Arabian peninsula. International Journal of Antimicrobial Agents 21: 153–157.
5. Memish ZA, Charrel RN, Zaki AM, Fagbo SF (2010) Alkhurma haemorrhagic fever a viral haemorrhagic disease unique to the Arabian Peninsula. International Journal of Antimicrobial Agents 36, Supplement 1: S53-S57.

## References

[1] Ftika L. and Maltezou H.C., Viral haemorrhagic fevers in healthcare settings. Journal of Hospital Infection, 2013 83(3): p. 185–192. 10.1016/j.jhin.2012.10.013 23333147

[2] Bray M. and Schaechter M., Hemorrhagic Fever Viruses, in *Encyclopedia of Microbiology* *(*Third Edition*)* 2009, Academic Press: Oxford p. 339–353.

[3] Lima E.Q. and Nogueira M.L., Viral Hemorrhagic Fever–Induced Acute Kidney Injury. Seminars in Nephrology, 2008 28(4): p. 409–415. 10.1016/j.semnephrol.2008.04.009 18620963

[4] Mohamed N.A., El-Raoof E.A., and Ibraheem H.A., Respiratory manifestations of dengue fever in Taiz-Yemen. Egyptian Journal of Chest Diseases and Tuberculosis, 2013 62(2): p. 319–323.

[5] Zeller H. and Georges-Courbot M.C., Les fievres hemorragiques virales. Antibiotiques, 2006 8(4): p. 215–220.

[6] Shibl A., Senok A., and Memish Z., Infectious diseases in the Arabian Peninsula and Egypt. Clin Microbiol Infect, 2012 18(11): p. 1068–80. 10.1111/1469-0691.12010 23066725

[7] Madani T., et al, Outbreak of viral hemorrhagic fever caused by dengue virus type 3 in Al-Mukalla, Yemen. BMC Infectious Diseases, 2013 13(1): p. 136. 23497142 10.1186/1471-2334-13-136 PMC3605114

[8] Van Kleef E., Bambrick H., and Hales S., The geographic distribution of dengue fever and the potential influence of global climate change. TropIKA Reviews (TropIKA.net), 2011: p. 22.

[9] Amarasinghe A. and Letson G.W., Dengue in the Middle East: a neglected, emerging disease of importance. Transactions of The Royal Society of Tropical Medicine and Hygiene, 2012 106(1): p. 1–2. 10.1016/j.trstmh.2011.08.014 22137535

[10] Ciccozzi M., et al, Phylogeny of Dengue and Chikungunya viruses in Al Hudayda governorate, Yemen. Infection, Genetics and Evolution, 2014 27(0): p. 395–401. 10.1016/j.meegid.2014.08.010 25183027

[11] Davies F.G., Risk of a Rift Valley fever epidemic at the haj in Mecca, Saudi Arabia Rev. sci. tech. Off. int. Epiz, 2006 25(1): p. 137–147. 10.20506/rst.25.1.1648 16796043

[12] GAS, Statistics Yearbook. 2010, General Authority for Statistics Saudi Arabia.

[13] CSOY, Statistic Yearbook 2013 of Yemen. 2013, Central Statistical Organisation of Yemen.

[14] Glyn Davies F. and Martin V., Recognizing Rift Valley fever—Food and Agriculture, in *FAO Animal Health Manual*. 2003, FOOD AND AGRICULTURE ORGANIZATION OF THE UNITED NATIONS: Rome.

[15] Shaif A., The epidemiology of Rift Valley Fever in Yemen and the risk of re-introduction from the Horn of Africa, in *Département des Maladies Infectieuse et Parasitaires*. 2011, Université de Liège: Liège p. 153.

[16] Memish Z., A., et al, Seroprevalence of Alkhurma and Other Hemorrhagic Fever Viruses, Saudi Arabia. Emerging Infectious Disease journal, 2011 17(12): p. 2316. 10.3201/eid1712.110658 PMC3311215 22172587

[17] Gubler D.J., Dengue and dengue hemorrhagic fever. Seminars in Pediatric Infectious Diseases, 1997 8(1): p. 3–9.

[18] Gubler D.J., Epidemic dengue/dengue hemorrhagic fever as a public health, social and economic problem in the 21st century. Trends in Microbiology, 2002 10(2): p. 100–103. 11827812 10.1016/s0966-842x(01)02288-0

[19] Gubler D.J., Epidemic Dengue and Dengue Hemorrhagic Fever: a Global Public Health Problem in the 21st Century, in *Emerging Infections* 1 1998, American Society of Microbiology.

[20] www.emro.who.int/docs/EM_RC54_5. Growing threat of viral haemorrhagic fevers in the Eastern Mediterranean Region: a call for action. 2007 [cited.

[21] Yacoub S. and Wills B., Dengue: an update for clinicians working in non-endemic areas. Clinical Medicine, 2015 15(1): p. 82–85. 10.7861/clinmedicine.15-1-82 25650206 PMC4954533

[22] Stanaway J.D., et al, The global burden of dengue: an analysis from the Global Burden of Disease Study 2013. The Lancet Infectious Diseases, 2016. 10.1016/S1473-3099(16)00026-8 PMC5012511 26874619

[23] Guzman M.G., et al, Dengue, Dengue Hemorrhagic Fever, in *International Encyclopedia of Public Health*. 2008, Academic Press: Oxford p. 98–119.

[24] Jimenez-Lucho V.E., Fisher E.J., and Saravolatz L.D., Dengue with hemorrhagic manifestations: an imported case from the Middle East. Am J Trop Med Hyg, 1984 33(4): p. 650–3. 6476211 10.4269/ajtmh.1984.33.650

[25] Fakeeh M. and Zaki A.M., Virologic and serologic surveillance for dengue fever in Jeddah, Saudi Arabia, 1994–1999. AmJTrop Med Hyg, 2001 65(6): p. 764–767. 10.4269/ajtmh.2001.65.764 11791972

[26] Zaki A., et al, Phylogeny of dengue viruses circulating in Jeddah, Saudi Arabia: 1994 to 2006Phylogénie du virus de la dengue circulant à Djeddah en Arabie Saoudite: 1994 à 2006 Filogenia del virus del dengue en Jeddah, Arabia Saudita: 1994 a 2006. Tropical Medicine & International Health, 2008 13(4): p. 584–592. 18248565 10.1111/j.1365-3156.2008.02037.x

[27] Jamjoom G.A., et al, Seroepidemiology of Asymptomatic Dengue Virus Infection in Jeddah, Saudi Arabia. Virology (Auckl), 2016 7: p. 1–7. 26917954 10.4137/VRT.S34187 PMC4758801

[28] Khan N.A., et al, Clinical profile and outcome of hospitalized patients during first outbreak of dengue in Makkah, Saudi Arabia. Acta Trop, 2008 105(1): p. 39–44. 10.1016/j.actatropica.2007.09.005 17983609

[29] Paolo R., et al, Imported Dengue Virus Serotype 3, Yemen to Italy, 2010. Emerging Infectious Disease journal, 2011 17(5): p. 929. 10.3201/eid1705.101626 PMC3321782 21529416

[30] Gamil M.A., et al, Prevalence of Dengue Fever in Jizan Area, Saudi Arabia. Journal of Pure and Applied Microbiology, 2014 8(1): p. 225–231.

[31] Al-Azraqi T.A., El Mekki A.A., and Mahfouz A.A., Seroprevalence of dengue virus infection in Aseer and Jizan regions, Southwestern Saudi Arabia. Trans R Soc Trop Med Hyg, 2013 107(6): p. 368–71. 10.1093/trstmh/trt022 23474472

[32] Giovanni R., et al, Co-circulation of Dengue and Chikungunya Viruses, Al Hudaydah, Yemen, 2012. Emerging Infectious Disease journal, 2014 20(8): p. 1351. 10.3201/eid2008.131615 PMC4111199 25061762

[33] Memish Z.A., et al, Is the epidemiology of alkhurma hemorrhagic fever changing?: A three-year overview in Saudi Arabia. PLoS ONE, 2014 9(2): p. e85564 10.1371/journal.pone.0085564 24516520 PMC3916301

[34] Memish Z.A., et al, Alkhurma haemorrhagic fever a viral haemorrhagic disease unique to the Arabian Peninsula. International Journal of Antimicrobial Agents, 2010 36, Supplement 1(0): p. S53–S57. 20800999 10.1016/j.ijantimicag.2010.06.022

[35] Rollin P.E. and Memish Z.A., Chapter 5—Alkhurma Hemorrhagic Fever, in *Emerging Infectious Diseases*. 2014, Academic Press: Amsterdam p. 61–71.

[36] Zaki A.M., Isolation of a flavivirus related to the tick-borne encephalitis complex from human cases in Saudi Arabia. Trans R Soc Trop Med Hyg, 1997 91(2): p. 179–181. 9196762 10.1016/s0035-9203(97)90215-7

[37] Madani T.A., Alkhumra virus infection, a new viral hemorrhagic fever in Saudi Arabia. Journal of Infection, 2005 51(2): p. 91–97. 10.1016/j.jinf.2004.11.012 16038757

[38] Madani T.A., et al, Alkhumra (Alkhurma) virus outbreak in Najran, Saudi Arabia: Epidemiological, clinical, and Laboratory characteristics. Journal of Infection, 2010 62(1): p. 67–76. 10.1016/j.jinf.2010.09.032 20920527

[39] Carletti F., et al, Alkhurma Hemorrhagic Fever in Travelers Returning from Egypt, 2010. Emerging Infectious Diseases, 2010 16(12): p. 1979–1982. 10.3201/eid1612.101092 21122237 PMC3294557

[40] Bente D.A., et al, Crimean-Congo hemorrhagic fever: History, epidemiology, pathogenesis, clinical syndrome and genetic diversity. Antiviral Research, 2013 100(1): p. 159–189. 10.1016/j.antiviral.2013.07.006 23906741

[41] Ergonul O., Chapter 10—Crimean-Congo Hemorrhagic Fever, in *Emerging Infectious Diseases*. 2014, Academic Press: Amsterdam p. 135–148.

[42] Watts D.M., et al, Chapter 67—Bunyaviral Fevers: Rift Valley Fever and Crimean-Congo Hemorrhagic Fever, in *Tropical Infectious Diseases* *(*Second Edition*)* 2006, Churchill Livingstone: Philadelphia p. 756–761.

[43] Ahmed J., et al, International network for capacity building for the control of emerging viral vector-borne zoonotic diseases: ARBO-ZOONET. Euro Surveill, 2009 14(12). 19341603

[44] El-Azazy O.M. and Scrimgeour E.M., Crimean-Congo haemorrhagic fever virus infection in the western province of Saudi Arabia. Trans R Soc Trop Med Hyg, 1997 91(3): p. 275–278. 9231193 10.1016/s0035-9203(97)90072-9

[45] Hassanein K.M., el-Azazy O.M., and Yousef H.M., Detection of Crimean-Congo haemorrhagic fever virus antibodies in humans and imported livestock in Saudi Arabia. Trans R Soc Trop Med Hyg, 1997 91(5): p. 536–7. 9463660 10.1016/s0035-9203(97)90014-6

[46] Hassanein K.M. and El-Azazy O.M., Isolation of Crimean-Congo hemorrhagic fever virus from ticks on imported Sudanese sheep in Saudi Arabia. Ann Saudi Med, 2000 20(2): p. 153–4. 17322717 10.5144/0256-4947.2000.153

[47] Leblebicioglu H., et al, Consensus report: Preventive measures for Crimean-Congo Hemorrhagic Fever during Eid-al-Adha festival. Int J Infect Dis, 2015. 10.1016/j.ijid.2015.06.029 26183413

[48] Al-Hamdan N.A., et al, The Risk of Nosocomial Transmission of Rift Valley Fever. PLoS Negl Trop Dis, 2015 9(12): p. e0004314 10.1371/journal.pntd.0004314 26694834 PMC4687845

[49] Chevalier V., Relevance of Rift Valley fever to public health in the European Union. Clin Microbiol Infect, 2013 19(8): p. 705–8. 10.1111/1469-0691.12163 23517372

[50] Davies F.G., The Historical and Recent Impact of Rift Valley Fever in Africa. The American Journal of Tropical Medicine and Hygiene, 2010 83(2 Suppl): p. 73–74. 10.4269/ajtmh.2010.83s2a02 20682909 PMC2913498

[51] Al-Afaleq A.I. and Hussein M.F., The status of Rift Valley fever in animals in Saudi Arabia: a mini review. Vector Borne Zoonotic Dis, 2011 11(12): p. 1513–20. 10.1089/vbz.2010.0245 21923257

[52] Anyamba A., et al, Rift Valley Fever potential, Arabian Peninsula. Emerg Infect Dis, 2006 12(3): p. 518–20. 10.3201/eid1203.050973 16710979 PMC3291449

[53] Balkhy H.H. and Memish Z.A., Rift Valley fever: an uninvited zoonosis in the Arabian peninsula. International Journal of Antimicrobial Agents, 2003 21(2): p. 153–157. 12615379 10.1016/s0924-8579(02)00295-9

[54] Flick R. and Bouloy M., Rift Valley fever virus. Curr Mol Med, 2005 5(8): p. 827–34. 16375716 10.2174/156652405774962263

[55] Trevor R.S., et al, Genetic Analysis of Viruses Associated with Emergence of Rift Valley Fever in Saudi Arabia and Yemen, 2000–01. Emerging Infectious Disease journal, 2002 8(12): p. 1415. 10.3201/eid0812.020195 PMC2738516 12498657

[56] Barry R.M., et al, Isolation and Genetic Characterization of Rift Valley fever virus from Aedes vexans arabiensis, Kingdom of Saudi Arabia. Emerging Infectious Disease journal, 2002 8(12): p. 1492. 10.3201/eid0812.020194 PMC2738526 12498669

[57] Al-Azraqi T.A., El Mekki A.A., and Mahfouz A.A., Rift Valley Fever in Southwestern Saudi Arabia: A sero-epidemiological study seven years after the outbreak of 2000â€“2001. Acta Tropica, 2012 123(2): p. 111–116. 10.1016/j.actatropica.2012.04.007 22569563

[58] Hotez P.J., Savioli L., and Fenwick A., Neglected Tropical Diseases of the Middle East and North Africa: Review of Their Prevalence, Distribution, and Opportunities for Control. PLoS Negl Trop Dis, 2012 6(2): p. e1475 10.1371/journal.pntd.0001475 22389729 PMC3289601

[59] eDEWS, Yemen: WHO Response to Dengue Fever Outbreak in Yemen Crisis 2015, in *Electronic Disease Early warning system*, WHO, Editor. 2015.

